# Rationale for recommending a lower dose of primaquine as a *Plasmodium falciparum* gametocytocide in populations where G6PD deficiency is common

**DOI:** 10.1186/1475-2875-11-418

**Published:** 2012-12-14

**Authors:** Nicholas J White, Li Guo Qiao, Gao Qi, Lucio Luzzatto

**Affiliations:** 1 Mahidol-Oxford Tropical Medicine Research Unit, Faculty of Tropical Medicine, Mahidol University, Bangkok, Thailand; 2 Research Centre for Qinghao, Guangzhou University of Chinese Medicine, Guangzhou, China; 3 Jiangsu Institute of Parasitic Diseases, Wuxi, China; 4 Istituto Toscano Tumori, Florence, Italy

## Abstract

In areas of low malaria transmission, it is currently recommended that a single dose of primaquine (0.75 mg base/kg; 45 mg adult dose) be added to artemisinin combination treatment (ACT) in acute falciparum malaria to block malaria transmission. Review of studies of transmission-blocking activity based on the infectivity of patients or volunteers to anopheline mosquitoes, and of haemolytic toxicity in glucose 6-dehydrogenase (G6PD) deficient subjects, suggests that a lower primaquine dose (0.25 mg base/kg) would be safer and equally effective. This lower dose could be deployed together with ACTs without G6PD testing wherever use of a specific gametocytocide is indicated.

## Background

Primaquine is an important but underused anti-malarial drug. The main limitation to the use of primaquine, either for the radical cure of *Plasmodium vivax* or *Plasmodium ovale* malaria, or as a gametocytocide in *Plasmodium falciparum* malaria, is the risk of haemolysis in patients who are glucose 6-phosphate dehydrogenase (G6PD) deficient [1,2]. The prevalence of G6PD deficiency, an X-linked trait, ranges from 5 to 20% in most malaria endemic areas of Asia and Africa, and in some communities it is even higher, so the total population at risk is very large [3]. Use of primaquine as a gametocytocide has great potential to reduce the transmission of falciparum malaria in low transmission settings, and in particular to help contain the spread of artemisinin resistant falciparum malaria in South-East Asia. The World Health Organization has recommended the addition of primaquine 0.75 mg base/kg (adult dose 45 mg) to artemisinin combination treatment (ACT) regimens for falciparum malaria in areas of low transmission [4], particularly in areas where artemisinin resistant falciparum malaria is a threat, “when the risk for G6PD deficiency is considered low or testing for deficiency is available” [5]. Unfortunately, testing for G6PDd is not widely available in malaria endemic areas. Thus concerns over the potential for dangerous iatrogenic haemolysis have limited the use of primaquine. As a consequence some countries deploy single dose primaquine as a gametocytocide, and some do not, and even when recommended, primaquine is often not given.

**Table 1:** **Recent WHO recommendation for “low dose” primaquine as a *P. falciparum *gametocytocide**[51]

In: (1) areas threatened by artemisinin resistance where single dose primaquine as a gametocytocide for *P. falciparum* malaria is not being implemented, and (2) elimination areas which have not yet adopted primaquine as a gametocytocide for *P. falciparum* malaria: A **single 0**.**25 mg base/kg primaquine dose** should be given to all patients with parasitologically- confirmed *P. falciparum* malaria on the first day of treatment in addition to an ACT, except for pregnant women and infants <1 year of age.

### Transmission-blocking effects

Transmission of malaria requires that a feeding anopheline vector mosquito ingests at least one male and one female gametocyte in a blood meal, and that the subsequent ookinete forms an oocyst in the wall of the mosquito gut which later matures to liberate viable sporozoites which migrate to populate the salivary glands. All the effective anti-malarial drugs, in addition to killing the asexual forms of all human malaria parasites, also kill early developing gametocytes (stages 1 to 3) of *P. falciparum*. Artemisinin derivatives reduce transmissibility substantially in acute falciparum malaria, largely by killing younger gametocytes (stages 1 to 4, and some early stage 5), but patients who have high numbers of infectious mature gametocytes at presentation may continue to transmit despite receiving ACTs [6-14]. The 8-aminoquinolines kill these mature infectious *P. falciparum* gametocytes rapidly [15]. Reduction in gametocytaemia is relatively easy to measure, and so has been used as an effect measure in trials, and has been the primary end-point in systematic reviews of transmission-blocking interventions. However, whilst there is a relationship between gametocyte densities and transmissibility, this relationship varies substantially between individuals [14-17]. In particular, patients with acute *P. falciparum* infections may have high blood densities of young stage 5 gametocytes which are not infectious. Furthermore drug effects on transmissibility precede effects on gametocytaemia, probably because of the lag between gametocyte killing and gametocyte clearance, so the transmission-blocking effect may be underestimated [15]. Dose–response assessment, therefore, requires direct assessment of the infectivity of patient's blood to anopheline mosquitoes. This is difficult, and in recent years has seldom been undertaken.

**Figure 1:**
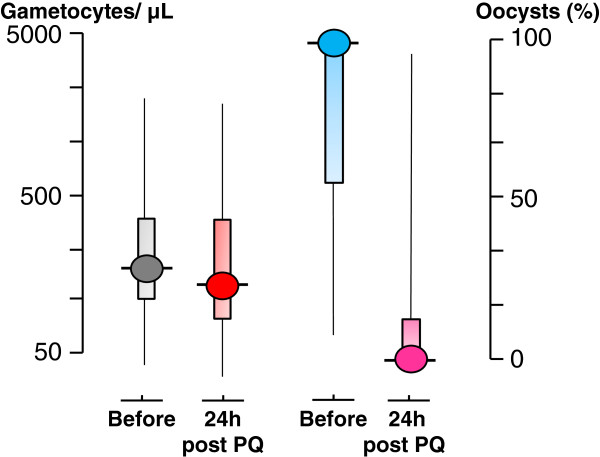
**The rapid sterilizing effect of primaquine in falciparum malaria; studies of mosquito infectivity following anti-malarial treatments of falciparum malaria with plasmoquine or primaquine in which oocyst assessments were made in mosquitoes which fed 24 hours after drug administration.** Oocysts were assessed typically in 10–20 mosquitoes 6–7 days after feeding Medians (IQR) and ranges shown. Gametocytaemia changed little in 24 hours (although it usually fell rapidly thereafter) (left side) (N=51), whereas oocyst numbers fell rapidly and were not found at all in the majority of mosquito batches fed on treated patients (right side) (N=79). When the fed mosquitoes were evaluated later sporozoites were correspondingly absent) [15].

The 8-aminoquinoline anti-malarial plasmoquine, the predecessor of primaquine, was discovered in 1924 [18,19]. Within two years plasmoquine had been shown to clear gametocytaemia in falciparum malaria [20], and between 1927 and 1929, in Panama, was shown to reduce rapidly the infectivity of *P. falciparum* to anopheline mosquitoes [21,22]. Even in these first studies it was evident that the transmission-blocking effect (assessed as reduction in mosquito oocyst counts) preceded the effects on gametocytaemia (Figure 1). Plasmoquine was commonly prescribed at adult doses of 60mg yet doses as low as 10mg provided rapid and potent transmission-blocking activity [21-25]. Primaquine replaced plasmoquine in the early 1950s as it was safer and more effective [15,26]. In clinical studies primaquine was three times more active against pre-erythrocytic stages, four to six times better in terms of radical curative activity against vivax malaria, and it was half as toxic. The transmission-blocking activities of the two drugs were never compared. In 1959, Martin Young reported on two gametocytaemic adult patients who received only 3 mg per day of primaquine alone and were non-infectious to anophelines within three and five days, respectively, of starting this treatment [27]. These observations suggested that doses much lower than the currently recommended adult gametocytocide dose of 45 mg would still be effective.

Individual data are available on 158 individual gametocytaemic subjects from studies conducted in different locations with different vectors and different drug exposures spanning approximately 80 years in which infectivity was assessed both by oocyst and sporozoite production 24 and 48 hours after drug exposure [15,28]. Of these 31 subjects received plasmoquine (before 1950) and 127 received primaquine (78 in hitherto unpublished studies conducted by Li Guoqiao and Chen Peiquan in Xuan Loc Hospital, Dong Nai Province, Vietnam, 1993–1996, and Kampong Speu Province Hospital, Cambodia, 2003–2004). In 65 of the patients primaquine was given together with an artemisinin derivative. These studies show clearly that both plasmoquine and primaquine rapidly and potently reduce the infectivity of *P. falciparum*. Primaquine increases the rate of gametocyte clearance but there is a lag phase of >24 hours before clearance accelerates. The effects on transmissibility assessed from mosquito oocyst numbers, and consequent sporozoite numbers and viability, occur much more rapidly than the effects on gametocyte densities (Figure 1). This suggests that most or all the gametocytes counted in blood films in the days following primaquine administration are dying or dead [15]. In these artificial infection experiments, the subjects selected for study commonly had high gametocytaemias, at the upper end of the distribution of gametocyte densities encountered in natural infections, and were highly infectious. This provided sufficient numbers for statistical assessment but it resulted in heavy mosquito infections (often > 30 oocysts per gut) which are rarely found in wildtrapped wildanopheline vectors. Drug effects on gametocyte viability are continuous, but the final effect on transmission to a mosquito is binary – it is either infected or it is not, and only one successful oocyst is all that is necessary for a mosquito to be potentially infective. For example, if there were 1,000 viable gametocytes/μL of blood, a reduction by 99% still leaves 10 gametocytes/μL - which may still be infectious; whereas if there were 10 gametocytes/μL initially, then a 99% reduction leaves an average density of 0.1/μL which is very unlikely to infect [15].

**Figure 2:**
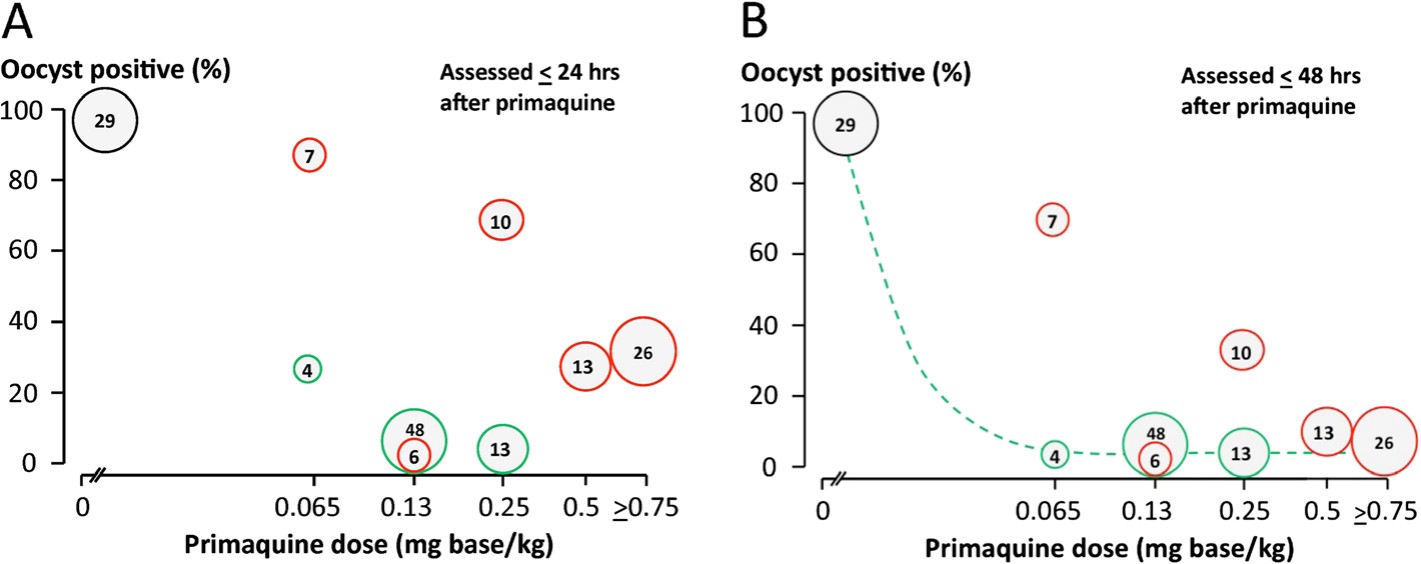
**Dose–response relationships for primaquine in reducing the infectivity of *P. falciparum *infected subjects to anopheline mosquitoes.** Vertical axis shows the proportion of fed anopheline mosquitoes which were infected. Pooled data from all studies conducted [15,28]. Left: Oocyst formation (proportion of patients who were still infectious to mosquitoes) from blood sampled 24 hours after primaquine dose, Right : Oocyst formation from blood sampled 48 hours after primaquine dose. Primaquine given with an artemisinin derivative is shown in green, and with a non-artemisinin derivative or no anti-malarial is shown in red. In these studies 29 patients received no primaquine. The size of the circle is proportional to the number of subjects in each group (shown within).

**Figure 3:**
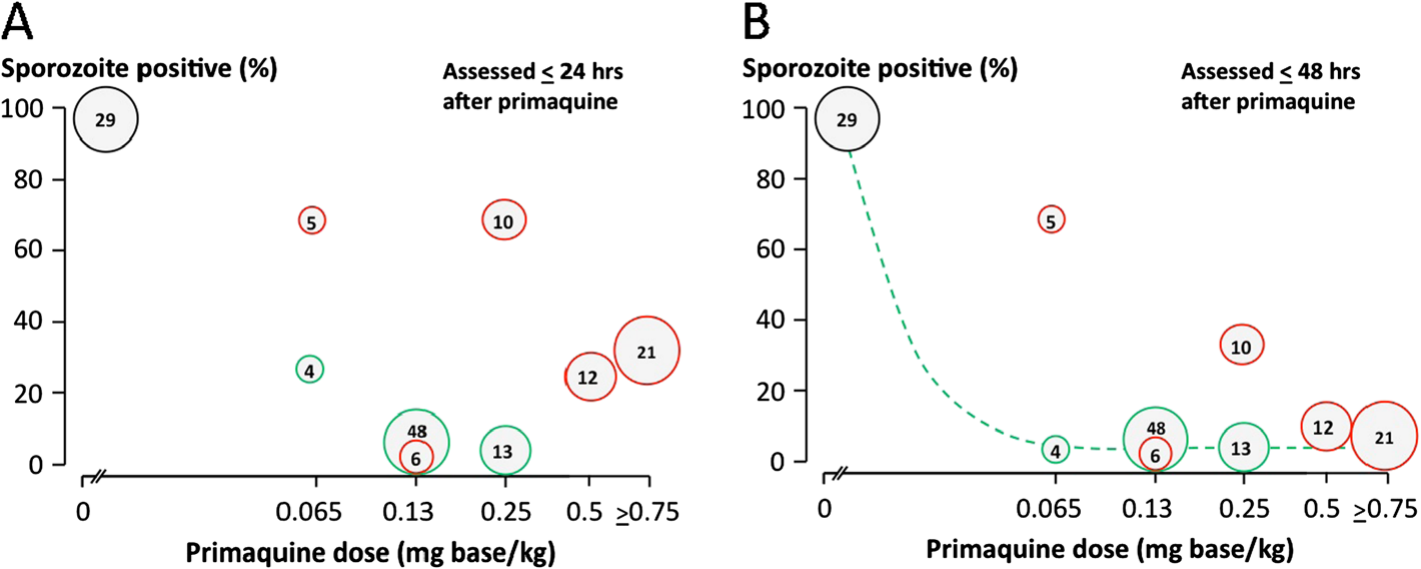
**Dose–response relationships for primaquine in reducing infectivity to anopheline mosquitoes as in Figure**2**, assessing sporozoite formation.** Left: Sporozoite formation assessed from blood sampled 24 hours after primaquine dose, Right: Sporozoite formation assessed from blood sampled 48 hours after primaquine dose. Numbers are not exactly the same as in Figure 2 because sporozoites were not assessed in all studies.

Compared to the volunteer studies, gametocyte densities in malaria infected individuals under natural conditions are generally much lower, with the majority being below the limit of routine microscopy detection [14,29]. Infected wild anopheline mosquito vectors when examined have correspondingly less intense infections (median 2 oocysts per gut) [30]. Thus these artificial infection studies in which man to mosquito infectivity was optimized tend to underestimate transmission-blocking drug effects at a population level. In the individual transmission-blocking assessments, nearly all adult subjects (102/108; 94.4%; 95% confidence interval 90.1 to 98.8%) who received primaquine at a dose ≥ 0.13 mg base/kg (adult dose ≥7.5 mg) had their infection sterilized within 48 hours after taking the drug. Oocyst (Figures 2 A and 2 B) and sporozoite (Figures 3 A and 3 B) responses were similar. Furthermore, concomitant treatment with artemisinin derivatives augmented the transmission-blocking effect significantly (Figures 2 A, 2 B, 3 A, 3 B). These dose–response assessments suggest a primaquine ED_90_ of approximately 0.06 mg base/kg given together with an artemisinin derivative, and 0.09 mg/kg without. This pooled analysis supports use of a dose lower than 0.75 mg base/kg (45 mg base adult dose) in transmission reduction strategies combined with an ACT.

### Safety

Primaquine is safe and generally well tolerated (with food) in G6PD normal subjects, although adult doses over 30 mg are associated with an increased incidence of abdominal discomfort [31]. On the other hand, in subjects who are G6PD deficient primaquine predictably causes acute haemolytic anaemia [32-34]. Over 180 different G6PD deficiency alleles have been identified [1,2]. Most of the mutations result in reduced enzyme stability: as red cells age their G6PD activity declines much more rapidly than in G6PD normal red cells, resulting in a marked reduction in the average enzyme concentration in the total population of circulating red cells. With many common G6PD deficiency alleles the mean enzyme concentration is less than 15% of that in normal red cells. G6PD deficient red cells are less able to regenerate NADPH, which is essential for maintenance of their principle anti-oxidant defences, particularly reduced glutathione, and for the function of catalase. Fava beans (which contain oxidant glucosides) and oxidant drugs (including sulphones and 8-aminoquinolines) produce predictable dose-dependent haemolysis in G6PD deficient subjects. Anaemia develops rapidly and in severe cases there may be nausea, vomiting, agitation, abdominal or loin pain, passage of dark coloured or black urine, and the patient may become jaundiced. Haemolysis can be life-threatening as a result of the anaemia itself, when it is very severe, or because massive intravascular haemolysis with haemoglobinuria may precipitate acute renal failure (more likely in adults). The peripheral blood film shows marked poikilocytosis, and characteristic abnormal red cells called hemighosts; Heinz bodies can be seen after appropriate staining. Methaemoglobinaemia also occurs: its mechanism is different, as it is not limited to G6PD deficient persons.

The severity of drug-induced acute haemolytic anaemia (AHA) in G6PD deficient persons depends on several factors, the most important of which are: (a) drug dosage, (b) the genotypic combinations at the G6PD locus (which are different in males *versus* females), and (c) the allelic variants of the G6PD gene.

### Drug dosage

#### Experimental studies with human volunteers

**Figure 4:**
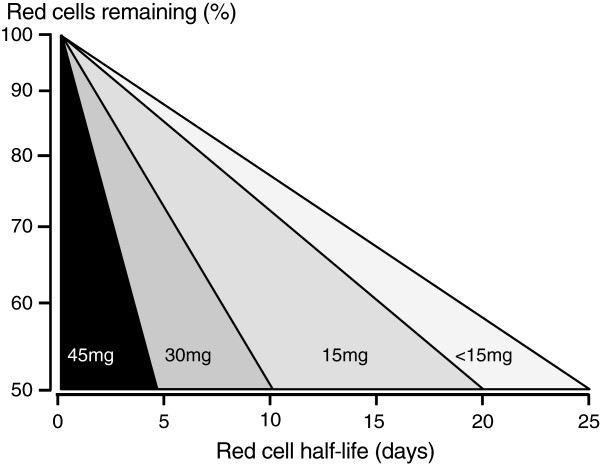
**Studies of **^**51**^**Cr labelled red cell survival in healthy adult hemizygous male volunteers with the A-variant of G6PD deficiency exposed to different dose regimens of primaquine in studies conducted by the University of Chicago-Army Medical Research Unit at the Illinois State penitentiary (Stateville) from 1950 to 1962.** Daily doses are shown within the range of red cell survivals that resulted. Daily administration of 45 mg base primaquine was considered to result in “dangerous haemolytic anaemia”, daily administration of 30 mg resulted in severe haemolysis and acute anaemia, and daily administration of 15 mg resulted in moderate haemolysis and mild anaemia [36].

**Figure 5:**
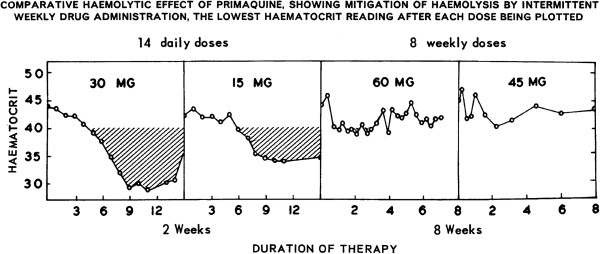
**Once weekly administration of primaquine 45 mg base for 8 weeks to healthy adult hemizygous male volunteers with the A- variant of G6PD deficiency resulted in less anaemia than daily administration of 15 mg for two weeks**[35].

Primaquine is given once when used as a *P. falciparum* gametocytocide, but it is given for 14 days for the radical cure of *P. vivax* and *P. ovale* malaria. When primaquine was administered daily to ‘primaquine-sensitive’ men (subsequently shown to have the A- variant of G6PD deficiency), there was brisk haemolysis with haemoglobinuria [31,32,35-37]. This haemolytic process was dose-dependent (Figure 4): haemoglobin fell by about one-third with 30 mg base primaquine daily, but by only ~15% with 15 mg primaquine daily. In contrast, a once weekly dose, even as high as 60 mg, had much less effect (see Figure 5). Primaquine is eliminated rapidly with a terminal elimination half-life of approximately five hours in patients with acute malaria, [38], so if primaquine is stopped the drug and its reactive metabolites are cleared quickly. This limits the degree of haemolysis. These dose–response observations in subjects with the African A- G6PD variant together with experience in large numbers of soldiers (see below) led to recommendation of a modified radical curative regimen in *P. vivax* and *P. ovale* malaria of 0.75 mg base/kg once weekly for eight weeks in patients with the A- type of G6PD deficiency [4,35].

#### Clinical case reports and series

When primaquine was first introduced in 1951 it was given in a radical curative regimen of 15 mg base per day for two weeks to prevent *P. vivax* relapse in some 250,000 US troops returning from the Korean war during their trans-Pacific sea voyage. Of these 10% were African-American soldiers [35]. It was stated that “there were very few serious adverse effects”, although later studies showed that in “primaquine sensitive” individuals the haematocrit often fell by one fifth! Weekly primaquine 45 mg (combined with chloroquine) was also used extensively in American soldiers in the Vietnam war. The net result of these clinical and experimental studies and the field experience was that a 0.75 mg base/kg (adult dose 45 mg) was recommended as the single gametocytocidal dose of primaquine in *P. falciparum* malaria.

No deaths have been reported following a single dose of primaquine [39]. Primaquine has been also used in the mass treatment of millions of patients in continuous regimens [40,41], mainly at the original adult dose equivalent of 15 mg base per day, without G6PD testing. All of the 13 attributable deaths reported (11 from severe haemolysis) resulted from repeated doses of primaquine [38]. The estimated risk of severe adverse effects (*i.e.* AHA) from this mass drug administration and routine treatment experience was approximately three per million. However, in prospective studies which focussed on safety, the estimated risk of severe adverse effects (severe anaemia, haemoglobinuric renal failure) was three orders of magnitude higher: 3.9 per thousand [39]. Although this figure still sounds reassuring at a population level it is important to emphasize that this risk is borne entirely by the sub-group of individuals who are G6PD deficient, all of whom will haemolyse with currently recommended primaquine doses to an extent determined by the severity of their enzyme deficiency [42,43]. These data also underscore the important difference in the safety of a single dose of primaquine *versus* continuous administration. The single 0.75 mg base/kg dose has generally been regarded as relatively safe [4,31] although in Vanuatu, where this dose of primaquine was introduced as a gametocytocide with the treatment of *P falciparum* malaria, over a period of two years seven cases of acute haemoglobinuria were observed with severe anaemia in G6PD deficient men and the policy was discontinued [44].

#### Genotypic combinations at the G6PD locus

G6PD is X-linked. Males have only one G6PD allele (they are either normal or deficient hemizygotes), whereas females have two (so they can be homozygous G6PD normal, or homozygous G6PD deficient, or heterozygous). Hemizygous males and homozygous females have full expression of G6PD deficiency: the level of G6PD activity in their mature red cells is nearly always ≤15% of normal values. Heterozygous females usually have only partial deficiency, and they have been referred to as having ‘intermediate’ deficiency. Among X-linked genes, G6PD belongs to the majority that are subject to X chromosome inactivation. Therefore ‘intermediate’ deficiency does not mean that each red cell has an intermediate level of G6PD: rather, it is a cumulative average, whereby some red cells have normal levels of G6PD whereas others are G6PD deficient. Moreover, because X-inactivation is a random event which takes place early in embryonic life (Lyonization), the resulting proportions of these two cell types are very variable. The severity of oxidant haemolysis in female heterozygotes will, therefore, vary from that in hemizygous males to that in G6PD normal persons (*i.e.* relatively little or no haemolysis) [42]. Unfortunately, in some studies heterozygous females have been labelled as G6PD deficient, and grouped together with G6PD deficient hemizygous males, which has compromised assessments of the clinical severity of acute haemolytic anaemia (AHA). The fall in haemoglobin in heterozygote females will be, on average, one-half of that seen in hemizygous male and homozygous females. From a clinical standpoint, heterozygotes with >70% G6PD normal red cells (*i.e.* < 30% G6PD deficient red cells) are unlikely to develop severe AHA.

#### Allelic variants of the G6PD gene

**Figure 6:**
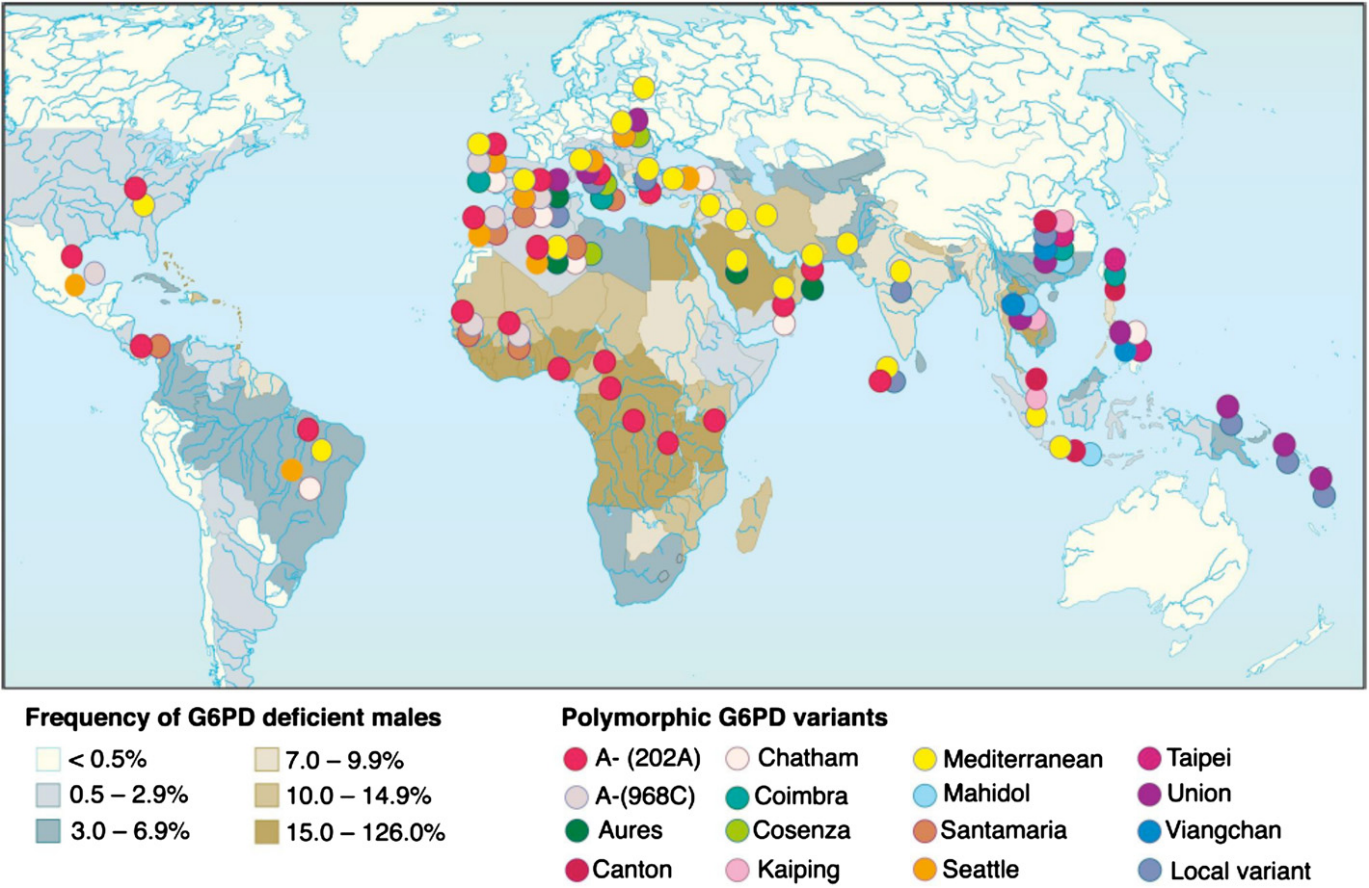
**Global distribution of polymorphic G6PD deficiency mutants**[47].

Many of the >180 G6PD deficiency alleles known are polymorphic, and are common in populations in the tropics (Figure 6). The degree of G6PD deficiency is an individual characteristic of each G6PD variant. For example the modal value of red cell G6PD activity is about 5% of normal for G6PD Mediterranean, whereas it is 13% for G6PD A-. This difference has clinical implications. In experimental studies carried out on otherwise normal subjects with G6PD Mediterranean the severity of AHA following primaquine was considerably greater [45,46] than with G6PD A- (see under (i) above). However, for both G6PD variants there is a sufficiently wide variation around the modal value of G6PD activity for the two distributions to overlap. Correspondingly, there is also overlap in the severity of the clinical manifestations. Many of the G6PD variants that are polymorphic in South-East Asia (e.g. G6PD Mahidol, G6PD Viangchan, G6PD Canton) have a modal level of activity which lies between that of G6PD Mediterranean and G6PD A-, which suggests that their haemolytic risk occupies a similar position.

Naturally the clinical consequences of drug-induced AHA depend to a considerable extent on clinical status when the drug is administered, and particularly on pre-existing anaemia [34,42,43,46,48]. Thus, the risk is markedly different in healthy adults, such as those who were given primaquine experimentally, or soldiers who were given primaquine as anti-malarial prophylaxis, compared to children with acute malaria in endemic areas who are often already anaemic.

Although more data are needed on the dose–response relationship for haemolysis following single dose primaquine in G6PD deficient subjects with different G6PD variants, the evidence available to date suggests that the risk of serious haemolysis is low. The data on the transmission-blocking dose–response reviewed here suggest that maximal responses are obtained with doses substantially lower than 0.45 mg base/kg, particularly if primaquine is used with an ACT. Thus, without attenuating the transmission blocking effect, in G6PD deficient individuals the risks of haemolysis could be reduced significantly by reducing the dose from 0.75 to 0.25 mg base/kg.

## Discussion

Incorporating a gametocytocide in a standard course of anti-malarial treatment may benefit the local community in terms of reduced incidence of malaria, and the global community by preventing the spread of resistant malaria parasites [5,17]. In areas of high or moderate transmission intensity this benefit is likely to be modest, because there is considerable redundancy in the transmission reservoir from asymptomatic individuals. On the other hand, in areas of low transmission every treated infection is a potentially important source of transmission, as well as of potential selection of drug resistant parasites. The degree of benefit depends on (i) the proportion of transmission derived from asymptomatic infections (ii) the proportion of symptomatic patients who receive gametocytocidal treatment (“coverage”) and (iii) on how early this is given in the course of a symptomatic infection (“access”). A single dose of primaquine (0.5 to 0.75 mg base/kg) is deployed as a *P. falciparum* gametocytocide by many countries, and is not deployed by others with similar epidemiological characteristics. Primaquine is practically never used in the private sector, which in some countries is the major provider of anti-malarial drugs. Although G6PD testing is encouraged, in reality it is usually not carried out. In the epicentre of artemisinin resistance in South-East Asia [49,50], where transmission-blocking strategies can play an important role in slowing the spread of resistant parasites [5], neither Cambodia nor Laos currently deploy primaquine as a gametocytocide because of safety concerns.

The wide disparity between the estimates of the frequency of primaquine induced AHA, derived mainly from passive reporting, *versus* the regular occurrence of AHA in prospectively studied G6PD deficient subjects is not surprising. Both anaemia and haemoglobinuria may go unnoticed or unreported, and the frequency of adverse events in G6PD deficient subjects is diluted by the much larger numbers of G6PD normal subjects in whom AHA does not occur. The importance of the different G6PD alleles has been probably over-emphasized. Although the degree of G6PD enzyme deficiency is less severe with G6PD A- than with most other alleles, AHA still occurs when primaquine is administered to individuals with this variant. With most other variants AHA can be expected to be more severe, and in the case of G6PD Mediterranean this is indeed the case [45]. On the other hand, clinical and experimental evidence shows that the haemolytic effect of a single dose of primaquine is considerably lower than with continuous administration [35]. The available data on haemolytic risks and transmission-blocking dose–response reviewed here suggest that a dose of 0.25 mg base/kg (15 mg adult dose) of primaquine in combination with an ACT would retain the transmission blocking effect of the currently recommended 0.75 mg/kg dose, whilst reducing substantially the severity of haemolysis in G6PD deficient subjects. This suggests that a 0.25 mg base/kg could be deployed without G6PD testing wherever use of a specific gametocytocide is indicated, a recommendation recently made by the World Health Organisation [51]. Meanwhile G6PD testing is still necessary for the seven to 14 day regimens needed for radical cure of vivax or ovale malaria, and so testing still needs to be made much more widely available (Table 1).

A recent Cochrane systematic review [52], which concentrated primarily on studies reporting gametocyte carriage rather than infectivity assessments, concluded that “primaquine should not be added to routine treatment of *P. falciparum* malaria with ACT until

1) it has been demonstrated that reducing infectivity of treated people in a variety of endemic situations reduces transmission on a community basis;

2) further research is done on safety and on adverse haematological effects for both G6PD normal and G6PD deficient subjects;

3) more is understood about the proportion of gametocyte carriers who present to receive treatment in a given population and time period

4) the cost of the policy balanced against the potential benefit is explored. In any case, patients should be screened for G6PD deficiency and those with variants predisposing to haemolysis should not be given PQ.”

These are large and difficult questions which would certainly take many years to answer if the detailed prospective clinical and epidemiological studies requested were undertaken. Whilst the Cochrane review [52] probably underestimated the transmission-blocking effect of primaquine by concentrating on gametocytaemia, it is true that there have been few trials providing evidence for community benefit. It is difficult to assess any single malaria control community intervention in isolation, and, even if such large cluster randomized trials were conducted, undoubtedly there would still be issues of generalizability. Currently however, there is an urgency to counter the spread of artemisinin resistance; and a number of countries are in pre-elimination and elimination phases so time is a very important commodity. The extensive data reviewed in brief here indicate that the safety of primaquine can be improved without loss of transmission-blocking efficacy. Primaquine is not being recommended as a gametocytocide in moderate or high transmission settings. While small foci of higher transmission with asymptomatic carriage are also features of low seasonal transmission settings, and more information on their epidemiology is needed, it is not clear how understanding more about them can be generalized to a treatment recommendation, particularly as there is such a diversity of epidemiological settings. Finally, any cost-benefit analysis must take into account whether control or elimination is the goal: in this respect, costs would be reduced and ease of deployment considerably enhanced if point of care G6PD testing was not necessary. In addition, there is also a critical cost, in terms of morbidity and mortality, of waiting years for more information and missing opportunities for decisive action [53].

Prospective studies should be conducted to confirm the safety of a single dose of primaquine 0.25 mg base/kg as a gametocytocide together with ACT in individuals with G6PD deficiency, and to assess transmission blocking activity dose–response relationships in different geographic areas, particularly in the context of artemisinin resistant falciparum malaria. In the meantime, based on the data reviewed above, there is sufficient evidence on safety and efficacy to support widescale deployment of a single dose of primaquine 0.25 mg base/kg as a gametocytocide without G6PD testing now where it is needed.

## Competing interests

The authors declare that they have no competing interests.

## Authors' contributions

NW conceived of the study, NW and GQ reviewed the transmission blocking studies, LGQ conducted the transmission blocking dose response studies with ACT, LL reviewed the haematological literature. All authors read and approved the final manuscript.
